# Learning experiences from an online QI fellowship programme during COVID-19 – a qualitative study

**DOI:** 10.1186/s12913-024-11590-z

**Published:** 2024-09-28

**Authors:** Richard A. Powell, Kandazi Sisya, Vimal Sriram, Rowan Myron

**Affiliations:** 1 Department of Primary Care and Public Health, School of Public Health, Imperial College London, London, England; 2 Ethnicity and Health Unit, Imperial College London, London, England; 3 Collaborative Learning and Capacity Building Theme, Faculty of Medicine, School of Public Health, NIHR Applied Research Collaboration Northwest London, Imperial College London, London, England; 4 Faculty of Medicine, School of Public Health, Imperial College London, London, England; 5 Director of Allied Health Professionals, University Hospitals Bristol and Weston NHS Foundation Trust, Bristol, England

**Keywords:** Quality Improvement, Leadership, Fellowships and scholarships, Problem-based Learning/Active learning, Distance Education, Communication

## Abstract

**Background:**

During the COVID-19 pandemic in the United Kingdom, multiple aspects of everyday human existence were disrupted. In contrast, almost all levels of educational learning continued, albeit with modifications, including adaptation to virtual—or online—classroom experiences. This pedagogic transition also occurred in the National Institute of Health and Care Research Applied Research Collaboration Northwest London’s (NIHR ARC NWL) Improvement Leader Fellowship, an annual programme focusing on quality improvement (QI). This qualitative study aimed to understand how these changes impacted the Fellows’ learning experience.

**Methods:**

We explored the experiences of two cohorts of programme Fellows (*n* = 18, 2020–2021 and *n* = 15, 2021–2022) with focus groups, analysed under a constructivist qualitative research paradigm.

**Results:**

The two primary and four sub-themes that emerged were: Online QI learning experience (benefits and challenges) and Implementing online QI learning (facilitators and barriers). While benefits had three further sub-themes (i.e., digital flexibility, connection between learners, and respite from impact of COVID-19), challenges had four (i.e., lack of interaction, technological challenges and digital exclusion, human dimension, and digital fatigue). While the facilitators had three sub-themes (i.e., mutual and programmatic support, online resource access, and personal resilience), barriers had one (i.e., preventing implementation and lack of protected time).

**Conclusion:**

Despite challenges to in-person ways of working, online learning generally worked for action-orientated QI learning, but changes are needed to ensure the effectiveness of future use of virtual learning for QI. Understanding the challenges of the translation of learning into action is crucial for implementation learning, gaining insight into how improvement Fellows navigated this translation when learning remotely and implementing directly in their workplace is key to understanding the evolving nature of implementation over the pandemic years and beyond.

**Supplementary Information:**

The online version contains supplementary material available at 10.1186/s12913-024-11590-z.

## Introduction

During the Coronavirus disease 2019 (COVID-19) pandemic in the United Kingdom (UK), multiple aspects of everyday human existence (e.g., work practices, sporting events, leisure activities) were suspended or disrupted to varying degrees between March 2020 and April 2022 [1]. In contrast, almost all levels of educational learning continued, albeit with modifications to the method of provision, including adaptation to virtual—or online—classroom experiences.

Virtual learning is “education being delivered in an online environment through the use of the internet for teaching and learning” [2]. A key feature is its lack of dependency on students’ co-location to learn and interact with educators and fellow students. The pandemic was not the first-time online learning was used. Indeed, it has been employed as a method of educational delivery in healthcare education for decades [3–6], with its benefits in enhancing student experience well documented [7–10]. Prior to the onset of the pandemic, however, the use of online learning had been inconsistent, possibly due to educators’ concerns regarding the additional burden of technology, including the lack of required technical support and resources [11]. The unprecedented challenges posed by the COVID-19 crisis resulted in the rapid transition to, and use of, online learning in healthcare education [12–14], often with minimal preparation.

This pedagogic transition also occurred in the National Institute of Health and Care Research Applied Research Collaboration Northwest London’s (NIHR ARC NWL) Improvement Leader Fellowship, impacting two cohorts of Fellows.

Initiated in 2010, the Fellowship is an annual programme, normally based around one day of face-to-face learning and three days of work-based learning per month, that focuses on quality improvement (QI). It is shaped by collaborative learning theory [15], where social interaction and multiple perspectives play an active role in fostering deeper thinking and learning [16]. Using a spiral curriculum—i.e., cyclical learning, increasing depth on each iteration, and learning by building on prior knowledge—Fellows learn in taught sessions then apply their learning to their QI projects within health and care settings.

This applied and action-focused pedagogy presented its own unique challenge when learning moved online. The Fellowship programme adopted online learning as a necessity (due to social distancing restrictions) imposed by the pandemic, with both educators and students forced to readjust, recalibrate, and innovate to accommodate online QI education [17]. Online learning was just one of the consequences of the pandemic; learners, many of whom were clinicians, were also under severe pressure during this time. With the spread of the pandemic, National Health Service (NHS) staff reported increased work-related stress and feeling a sense of responsibility to protect others, which negatively affected their mental health and wellbeing [18–20]. Recent research has highlighted the impact of COVID-19 on medical education and the advantages of using technology to facilitate such education [21]. Lessons learned from virtual training [22] highlight the positives of virtual education and how it can facilitate application of knowledge. Given the rapid change in healthcare education delivery following the pandemic’s onset, gaps exist in our understanding of how these online pedagogic changes impacted healthcare professionals’ learning experience. More particularly, how this affected the implementation of action-focused learning, such as the Fellowship. This study therefore aims to address this knowledge gap by exploring the experiences of online learning among participants on the NIHR ARC NWL Improvement Leader Fellowship in the context of QI education and learning.

## Methods

### Philosophical position

This research took a relativist ontological position, with a constructivist epistemological position, theorising that ‘truth’ is not absolute but is created by each individual and influenced by the context of that individual [23]. Hence, the research sought to understand how each participant constructed their experience of online learning and the application of that learning during the Fellowship. We followed recommendations from Consolidated criteria for reporting qualitative research (COREQ) in structuring this manuscript (Additional file 1).

### Participants

**Fig. 1:**
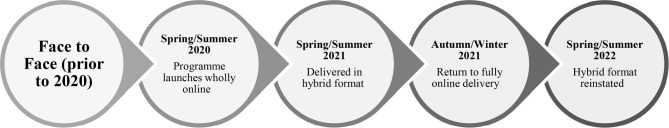
Timeline showing changes to NIHR ARC NWL Improvement Leader Fellowship for cohort 2020-21 and 2021-22

We explored the experiences of two cohorts (*n* = 18, 2020-21 cohort and *n* = 15, 2021-22 cohort) of NIHR ARC NWL programme Fellows. The first cohort received QI education between May 2020 and April 2021; the onset of COVID-19, and the associated governmental public health restrictions (e.g., social distancing, work from home orders), meant Fellows were required to learn almost exclusively online, with occasional meetings held in a hybrid format towards the end of the Fellowship. The second cohort received QI education between July 2021 and April 2022; by the summer of 2021, public health restrictions were easing, and the Fellowship was mostly delivered in-person, but Fellows could attend meetings online if they needed to isolate (as a result of an infection or to protect a vulnerable person). However, with the increase in COVID-19 cases and hospital admissions in Autumn/Winter 2021, the Fellowship was moved entirely online in January 2022 until March of that year, when it returned to a hybrid format (Fig. 1).

All purposively sampled Fellows were notified of the planned focus group [24] discussions in advance in an emailed information consent sheet, but their purpose was reiterated both verbally on a face-to-face basis on the day and in writing by the course leader (RM) before data collection started. RM, VS and KS had a pre-existing relationship with the Fellows as course staff members; RP was introduced to the second cohort, with his background, research experience and interest in undertaking the research explained. No Fellows withdrew from the study; there were a small number (1 in 2022) that could not attend on the day for practical reasons. All participants were offered the opportunity to ask any questions before providing their informed written consent.

### Research team, reflexivity and integrity

The focus groups were conducted by RP, a male employed at the time of the study as an Evaluation and Project Manager, who has two health-related Masters degrees and extensive experience of writing on and conducting qualitative research and by a colleague with experience in qualitative research and discussion group facilitation who was not involved in the completion of the study. As this study involved qualitative data collection, analysis and interpretation, we considered the authors’ assumptions and preconceptions regarding the phenomenon of interest (i.e., a QI educational programme delivered online). The authors come from different professional backgrounds (occupational therapist, psychologist, social scientist) with varying levels of experience in education and QI; this helped minimise any potential influence of individual researcher’s conceptions and preconceptions regarding the phenomenon of interest. This research process was conducted in collaboration with all the authors who acknowledge they have clinical and non-clinical backgrounds and levels of expertise in teaching, using digital education methods and QI. Additionally, the data collection and analysis were conducted by members of the research team who have declared no conflict of interest. As a result, the research is not affected by external influences. The use of standard approaches to data collection, analysis and interpretation as well as author reflexivity minimised the impact of the research team’s preconceptions [25]. Reflexivity and integrity were maintained throughout this research study [26].

### Data collection and management

Four approximately one-hour, focus groups were held by facilitators in an academic setting, with field notes made immediately after. The focus group discussion guide was developed for this study (available in supplementary files). Active participation of all group members was ensured by a pre-discussion outlining of the rules governing interactions (e.g., being respectful of others, including opportunities for speaking) and an inclusive engagement approach that actively sought opinions from less vocal participants. Discussions were recorded using Zoom software, from which transcribed documents were automatically generated. Both cohorts followed the same study discussion guide, which was adapted in the course of discussions for question order and supplementary queries as needed. Key areas covered by the guide were participants’ learning experiences, their use of this learning within health and social care settings—and any facilitators and barriers—how this could be improved, differences, and potential future differences, to how they work, including with colleagues, and the impact of COVID-19 on their learning experience - its challenges and benefits - and project implementation. Data saturation is usually used in qualitative research to ascertain sample size, however there are differences as to when and how this is achieved. Evidence from a review of saturation in focus groups [27] suggests that 90% of saturation can be reached by conducting 4–5 focus groups. After four focus groups we did not perceive that additional new ideas would add further useful insights for this research study.

### Data analysis

The verbatim transcripts produced by the Zoom platform were manually checked for errors by KS and corrected. We employed the template analysis approach to thematic content analysis [28]. An initial coding template was developed manually by RP, employing marker pens to highlight and code textual segments relevant to the study aim. Specifically, we aimed to understand the impact of restrictions imposed as a result of COVID-19 on the learning experience as perceived by participants. We used provisional a priori codes derived deductively from the focus group guide anticipated to be especially relevant to the analysis and augmented them inductively from the transcripts. These codes were arranged hierarchically, using broad umbrella themes followed by narrower sub-themes. This coding tree was subsequently independently and critically reviewed by KS against the transcripts. Following the addition of a small number of new variables and the recoding and regrouping of some initial codes, a consensus was achieved. Exemplar quotes were then selected to illustrate the thematic findings, and a final report of themes and sub-themes generated.

### Ethical approval

Ethical approval (reference number: EERP 1920-082a) was granted by the Imperial College London (IC) Research Ethics Committee. Written informed consent to participate was obtained from all participants in the study.

## Results

The focus groups included 18 (2020-21 cohort) and 15 Fellows (2021-22 cohort), comprised of clinical practitioners (see Table 1).

**Table 1:** Breakdown of participants’ socio-demographic characteristics

	Group 1	Group 2	Group 1	Group 2
	2020-21	2020-21	2021-22	2021-22
Socio-demographic characteristics	**n**	**n**	**n**	**n**
Sex				
* Male*	2	2	1	1
* Female*	4	10	6	7
Professional Fellow^a^	6	11	6	7
Patient Fellow^b^	0	1	1	1
Total	6	12	7	8

^a^A professional fellow is defined as someone who is paid by a healthcare organisation to provide care

^b^a patient fellow is defined as someone who is/has undergone treatment and then volunteers, is unpaid for their work for the NHS (this includes carers)

**Fig. 2:**
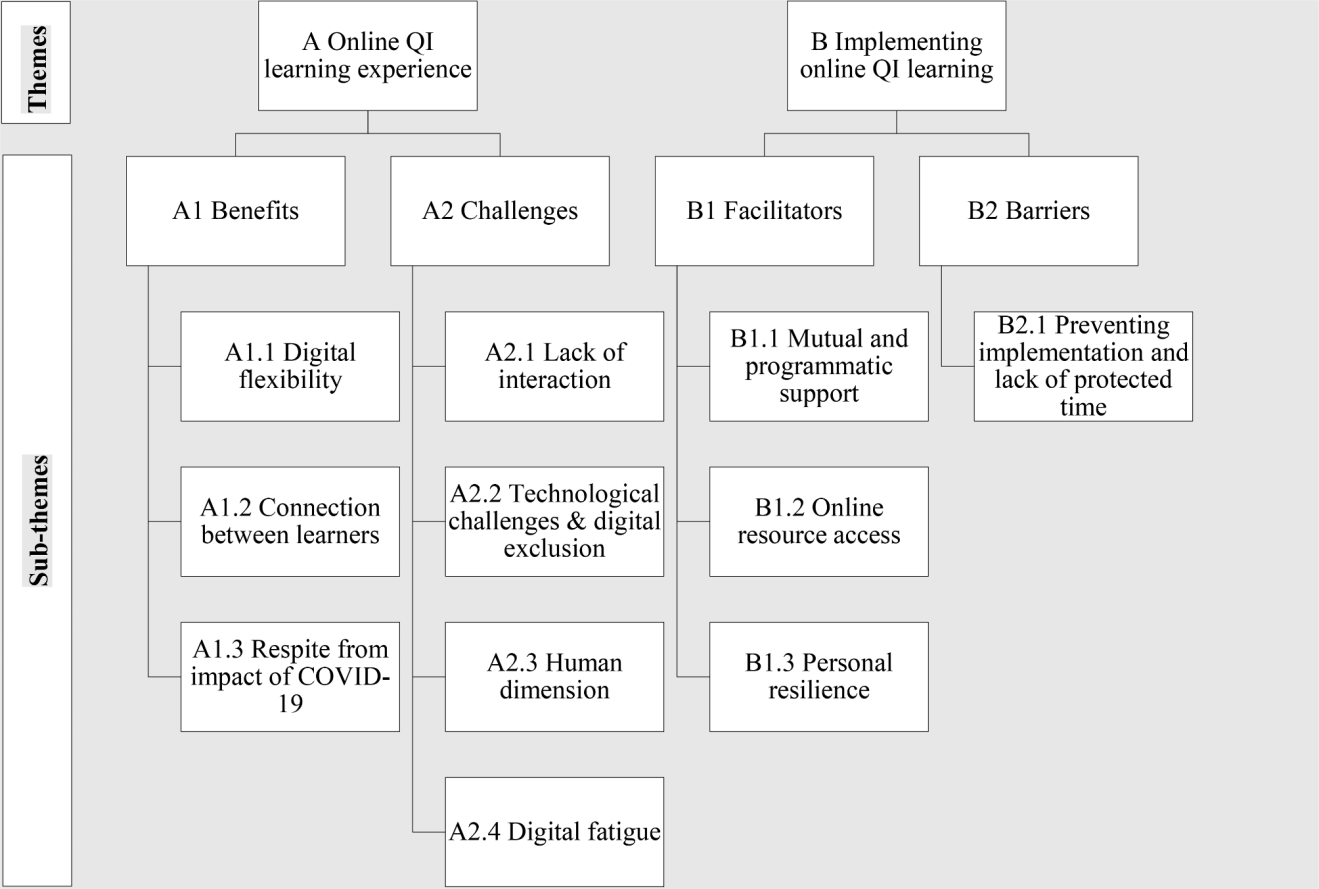
Themes and sub-themes identified through thematic analysis

Analysis of data showed two primary and four sub-themes: Online QI learning experience (benefits and challenges) and Implementing online QI learning (facilitators and barriers). While the benefits under the online QI learning theme had three further strands (i.e., digital flexibility, connection between learners, and respite from impact of COVID-19), the challenges had four further strands (i.e., lack of interaction, technological challenges and digital exclusion, human dimension, and digital fatigue). While the facilitators under the implementing online QI learning had three further strands (i.e., mutual and programmatic support, online resource access, and personal resilience), the barriers had one further strand (i.e., preventing implementation and lack of protected time) (see Fig. 2). The following section elaborates upon these findings. Additional illustrative quotes are provided in Table 2.

**Table 2:** Additional illustrative quotes

Theme	Sub Theme	Example Quote1	Example Quote 2	Example Quote 3
Online QI learning Experience	Benefits	On one occasion I wasn’t too well. Couldn’t come in, but I’ve benefited by joining it [Fellowship day] from home. So that was a positive in itself. (Group 1: 2021-22)	… often as professionals we feel constant guilt, we’re not doing enough here, we’re not doing enough there and with our personal life… it’s a really important point, that your flexibility has sort of took some of that pressure away and allowed us to try and balance the varying demands on our time. (Group 1: 2020-21)	We would frequently break out into Zoom, small groups, and I would get to hear kind of what other people do, so that was really helpful. (Group 2: 2020–21)
	Challenges	I think one of the negatives for me, is that the temptation when it is at home, is to not completely clear your diary. It becomes easy to try and think you can multitask and oh yes, I can just nip out for that lunch time meeting on Teams or kind of super quickly check my emails (Group 1: 2020-21)	The networking was a little bit harder. We were talking about this at lunch, the bits that we missed out on, going for lunches, drinks, pizza. Actually, a lot of the collaborative relationships are built in those times. Not just when you’re listening to the content of the tuition, or the didactic part of it. (Group 1: 2020-21)	So actually, these days of attending in-person have been like gold dust to us. Where it’s time to set aside that we could actually spend together … but even with … the virtual learning, we tried to replicate that, we tried as far as possible when we weren’t attending in person to all meet in one space. I think we were more distracted, (Group 2: 2021-22)
Implementing online QI learning	Facilitators	But I think the other challenge for me has just been the culture of the unit… I think QI, change transformation, in a culture that doesn’t like to change… And I think it’s really difficult as individuals… you feel like a small fish in a very big pond and that’s quite difficult. (Group 1: 2020-21)	Having those resources that you can go to, that are quite easily accessible. I think that’s the only thing maybe that when I think about starting this project again, hopefully come autumn- where do I find all these resources again … (Group 2: 2020-21)	… even if it’s a small catch up, we feel heard and there is a moment for us to share the struggles, or to share different approaches or you know, just to brainstorm within the team and having you know … So, it’s just a moment to feel heard and engaging so we keep the motivation going within the hard times. (Group 1: 2020-21)
	Barriers	And I think that’s been for us the biggest hurdle, we started at a point where we already had some service in place that we wanted to build upon. But actually, the service we had was taken away during the Fellowship. So, then it’s completely changed our project and our approach and having dedicated time to do it with kind of other NHS pressures it’s been really hard. (Group 2: 2021-22)	I just sort of squeezed in if I found any time. Like there wasn’t time to do it [QI project] allocated from my managers. So, I think that the time when you actually come on the day as well, if you can do anything when you’re there I’ve found that really helpful to be like quick, get some, some work done. (Group 1: 2021-22)	We all have Zoom, um, fatigue, you know, a full day by Zoom. It’s just too much. So, either you think about it very well and you kind of make it as close to real or you’re missing a lot. (Group 2: 2020-21)

### Online QI learning experience

#### Benefits

Participants identified benefits from the online learning for QI including ability to better manage their time and decrease the need to travel to a learning venue.

##### Digital flexibility

Whilst acknowledging the potential downside associated with digital learning, participants recognised and valued the flexibility the medium afforded them.

*There are pros and cons … it’s easy to dial in … it’s convenient*,* it’s quite helpful. You know*,* on a personal level*,* husband working away*,* kids being picked up and dropped off. It makes that element of things way less stressful. (Group 1: 2020-21)*

For one participant, this flexibility was replicated in delivery of healthcare to patients online as part of their QI project.

*COVID has revolutionised the face validity of digital health. Because before … I mean*,* in my practice*,* you know*,* before if I’d have said I can do this assessment over Zoom or whatever*,* people would have been like*,* ‘Ooo*,* that can’t be as good. Now*,* everybody’s like*,* ‘Oh*,* no problem’…’ (Group 2: 2020-21)*.

##### Connection between learners

Participants noted that the connection tying them together was critical as co-learners—partly to alleviate anxieties—and this was not broken by the challenge of online learning, partially by breaking up discussions into smaller interactive groups.

*So, as long as we keep connected*,* I think virtual or not*,* it’s not the distance*,* it’s the communication that brings us together*,* isn’t it? It’s not the distance. We can be in different places and different countries now*,* isn’t it? And we still link and connect. Sharing experiences*,* I think is the main thing. (Group 1: 2020-21)*

##### Respite from impact of COVID-19

The continuation of the Fellowship online additionally offered participants respite from the vagaries of the pandemic and provided a stable framework that maintained a focus on their studies.

*… it was nice to dial into the specific sessions. People could have a bit of a break from everything else that was going on and that actually*,* a bit of a light at the end of the tunnel moment. (Group 1: 2020-21)*

This opportunity to continue learning online or in a hybrid format provided continuity and assurance of meeting at regular intervals.

*Learning-wise was probably the discipline of having regular times to meet and reflect on … one of the biggest benefits for me was in having those moments … in the diary to come together and then being … put on the spot and asked*,* you know*,* ‘what have you learnt since last time?‘*,* ‘what have you done differently?‘. Actually*,* forced me to reflect on that*,* which that was very helpful for me. (Group 2: 2020-21)*

#### Challenges

##### Lack of interaction

This sub-theme relates to the challenges raised by online environments. One aspect of the learning process missed by Fellows was the intimacy of personal encounters, as well as ‘being present’, especially in the context of QI learning, which was usually delivered through in-person interactive elements.

*I have really valued coming in today and having it face-to-face. On Zoom*,* it’s just not a conversation. It’s one person talk[ing] and then you don’t get people feeding into you. You can see some nods*,* but you don’t get the … you know*,* the validation or*,* yes*,* kind of conversation. (Group 2: 2020-21)*

Online interactions also were seen as being ‘unnatural,’ in the sense that they removed the spontaneity of exchanges:

*If you’re on Zoom and everyone’s muting and unmuting*,* it becomes really difficult to have sort of like a natural discussion about things … you get so much more*,* that just comes out [of] something spontaneous that someone comes up with. (Group 1: 2021-22)*

Additionally, it was the nature of the interaction between Fellows that was noticeably absent; the ‘softer’, less formal interactions that facilitate interpersonal connections between learners.

*To the conversation we spoke about earlier*,* the softer skills*,* it’s the coffee breaks*,* the corridor conversations*,* the informal stuff that you have that actually really makes connections*,* makes relationships work and not having that over a screen just makes that 100 times more difficult*,* I think. (Group 1: 2020-21)*

This lack of informal interaction was potentially less problematic for those working in clinical care positions who, attending work in, for example, hospital settings, were “*able to have those corridor conversations and … speak to people*,* my colleagues*,* in person*” *(Group 1: 2020-21)*.

Additional to making connections, participants spoke of the value of collaborative learning.

*Really*,* I think if I’m honest with myself*,* you get much more out of it being in the room. You’re more attentive. You take more away. It’s easier to have small group discussions. It’s easier to do the group work. So yeah*,* I suppose ultimately less convenient*,* but definitely better if you are here. And I think it’s better than if it’s just all offline. (Group 2: 2021-22)*

##### Technological challenges & digital exclusion

Some participants noted the technical challenges to learning that can exist using digital platforms. Those can both be from the learners’ perspectives, but also from the facilitator’s viewpoint:

*And then you’ve got somebody who has internet connection issues*,* you know. Not everyone is sort of up to speed with all this Zoom*,* Teams and going in and then. You know*,* even until today I can’t do a sort of like background drop thing. (Group 1: 2021-22)*

This was also true for hybrid sessions (in-person learning session with some participants joining online)

*90% of the time*,* you can’t hear if you’re on virtual and it’s going on in person*,* you don’t know what’s happened on that day. You can’t hear people that are facilitating*,* and you can’t hear people in the room. (Group 2: 2021-22)*

For others, it was less the technological challenges of digital learning as it was about digital exclusion, especially for some Patient and Public Involvement (PPI) representatives, both for learning and as users of the NHS.

*Regarding COVID*,* you know*,* the digital - I’m all for that but it has got its disadvantages from PPI perspective actually. Many people don’t have connections*,* cannot afford [it] … now where possible*,* [the] NHS [is] pushing for the digital appointments and things like that*,* and I think that is going to be a sort of sad case because already local authorities [regional organisations] and everywhere assuming you … have digital access. (Group 2: 2020-21)*

##### Human dimension

This sub-theme relates to the many human aspects of learning in an online environment. Participants lauded this human aspect of their interactions that virtual learning could not provide to a comparable extent.

*I just don’t think that we should allow technology to replace human connection because we are social beings … I’d rather*,* you know*,* not attend at all if I couldn’t come because for me*,* the Zoom was very difficult … I don’t think we should allow technology to take over all our lives. That’s totally disastrous. (Group 1: 2021-22)*

##### Digital fatigue

Another negative linked with online learning was the associated tiredness, which can result in distraction and disengagement.

*I think sometimes when I’m on online it’s a lot easier to get distracted*,* or lose concentration or just switch off … sometimes I feel a like I just zone out after a while*,* because I’ve sat there and I’m looking at the screen and not trying to take it all in. (Group 1: 2021-22)*

### Implementing QI learning

This theme refers to the implementation of learning for the fellows in their QI projects in the workplace. We consider the facilitators and factors that support or inhibit implementation, which was part of the curriculum for QI learning.

#### Facilitators

##### Mutual and programmatic support

In some ways, peer support was viewed as beneficial to alleviate the sense of ‘helplessness’ initially caused by the pandemic and further support was derived from the programme itself.

*Yes*,* so I’ve had quite a difficult experience… because I’ve experienced so many systemic delays… However*,* the Fellowship has reassured me a great deal that this is quite common and*,* and that there’s a lot of learning in the delays. And I also think I’ve retained quite a lot of the learning around PDSA [Plan*,* Do*,* Study*,* Act] cycles and things like that. And I’m quite confident that I’d be able to apply now I’ve got through the barriers. (Group 2: 2021-22)*

##### Online resource access

The transfer of the Fellowship programme’s educational materials to bespoke online platforms, including an e-learning platform (QI4U) and cohort-specific website, was seen as very helpful.

*I found the QI4U really helpful and going back to that after a little off time as well was really good. (Group 2: 2020-21)*

##### Personal resilience

Individual Fellows discussed the role of their personal resilience in moving forward from the pandemic, and how this contributed to the implementation of online QI learning.

*There is always going to be barriers in an improvement project*,* there is always going to be challenges … exactly how we still keep moving forward and have the kind of expectations. I think the expectation in our group is*,* ‘COVID’s got in the way*,* oh we’ve all struggled*,* it’s been a really difficult time’. As it completely has*,* but how you start to move that narrative forward- that’s where our team will be able to start to get that ball rolling. (Group 2: 2020-21)*

#### Barriers

##### Preventing implementation and lack of protected time

In addition to affecting classroom learning, COVID-19 affected Fellows’ QI projects as well. Project progress was tethered to their QI learning and implementing in practice what they were learning as part of the ‘spiral curriculum’. The pandemic forced staff to redeploy from their usual roles to address the urgent needs of patients, so their QI projects and ambitions suffered, even if some of the lessons learned through the Fellowship programme could be applied to their new positions.

*So*,* my original project was in a compassion focused-therapy programme for patients*,* but I went into employee health and was doing stakeholder mapping*,* was doing process mapping*,* was drawing a lot for the stuff that I’ve learned [from the course] into that environment but not necessarily knowing that I was doing it. (Group 2: 2020-21)*

One of the common complaints among Fellows was that during the pandemic, they did not have access to protected time for their QI projects, with their line managers not allowing them to dedicate time to action their learning, which (if they had protected time) would have resulted in additional implementation-based learning.

*We had to do all of that through COVID where actually my*,* my schedule increased three times during COVID. I was asked to work three times more … and on top of that*,* you know*,* the Fellowship and on top of that*,* not having protected time for research. (Group 1: 2021-22)*

## Discussion

Recent literature has highlighted the benefits of online learning in healthcare education, including increased accessibility and reach, greater flexibility in delivery, and financial and time savings [29–31]. However, online learning has also faced challenges. Examples include technical problems, such as healthcare educators’ and learners’ familiarity and competence with tools and access to digital infrastructure, the unknown impact on student outcomes as educators experience difficulties maintaining students’ engagement and lack of speaker continuity, and its socio-emotional impact, such as loss of social connections, collaborative learning, and poor student wellbeing [32–35]. The recent findings on the impact of COVID-19 on medical education and the advantages of using technology to facilitate such education gives a key indicator of how healthcare educators can move forward in utilising the positive aspects of virtual education, whilst working to mitigate some of the disadvantages identified in this study [21, 22, 36].

This study of online QI learning found participants place value on face-to-face interaction due to the richness of learning in person. However, there is also a clear indication that where face-to-face learning is not possible, there are key ways in which online education can provide an enriched space for collaborative learning. This study’s participants highlighted problems in the transition from face-to-face to virtual learning, particularly for QI learning, which is traditionally taught in person, is discussion based and ‘hands-on’. Especially problematic were technological aspects (e.g., poor audio), the lack of interaction, technological challenges and digital exclusion, the human dimension of the learning experience and digital fatigue. Some of these challenges exacerbated existing challenges to full-time staff finding, for example, the ringfenced time to learn and then implement their QI projects.

However, there were positives arising from virtual learning that were also reported by participants. These included the digital flexibility, the connection between learners and respite from the impact of COVID-19 on other professional and personal aspects of their life. These are equally important to note given in the post-pandemic landscape, there is a possibility that healthcare educators, like clinical healthcare providers, may adopt some of the innovations initiated during the height of the pandemic, including a hybrid approach to training [30, 32].

In terms of implementation and collaboration, participants described a difference between their online learning experience and the impact of COVID-19 on their learning. There was awareness of a relief in coming together, a ‘light at the end of the tunnel’, which the regular ‘headspace’ of the learning days appeared to provide. Additionally, this particular type of learning was seen as collaborative, with group discussions often actively focussed on a common problem (e.g., action learning sets) [37]. The active process of solving a problem together possibly relieved some of the helplessness reported by many during COVID-19 lockdowns [38]. The ability to actively apply what they learned, not only in the day, but to take that learning back into practice was also seen as valuable. A positive aspect of the Fellowship is that despite learning moving to an online platform, Fellows were still able to implement change in their healthcare settings and described an ability to connect with each other that added to their experience. The pedagogy of the Fellowship (i.e., collaborative learning theory and social constructivism) [14, 35] is designed to support implementation and action from learning. It is also designed to foster collaboration across inter-disciplinary and indeed professional boundaries [16], and some of this collaboration can and indeed did happen even in the absence of physical face-to-face meetings and, where possible, through hybrid meetings [39].

To help others designing and implementing learning programmes for QI, we also share information that could have supported learners. The Fellowship programme itself implemented some of the QI methodology to make changes and adapt during the course of the programme to facilitate and better integrate online learning. Online learner feedback enabled the faculty to implement quick and cost-effective solutions. To overcome difficulties encountered during hybrid sessions as pointed out by participants, an addition of a lapel mic ensured audio could be clearly heard online and, in the room, a portable webcam ensured visual engagement regardless of venue. The programme also funded access to a laptop for a patient fellow who was digitally excluded. Solutions do not always have to be large and expensive [37]. Communicating this to fellows through modelling of good behaviour and resolving ‘problems’ within the learning environment in an agile and quick manner encouraged fellows to transfer and apply similar techniques within their own workplace.

Lastly, it is important to note the study’s strengths and limitations.

This is one of a few studies qualitatively examining the pedagogic experiences of adult learners during the COVID-19 pandemic and to our knowledge the first study that specifically reports on QI learning delivered through online and hybrid formats. It provides useful insights into the use of online learning, highlighting positive and negative aspects that can help inform flexible and improved models of hybrid learning that are responsive to the needs of individual learners. However, due to the self-selected nature of the sample, participants’ experiences may not be representative of those of the Fellowship learning community during the same time period. Additionally, given the exploratory nature of this study, it did not enable comparison in experience between the two learner cohorts. In this regard, it is important to note that differential delivery modes of learning between the two cohorts may mean that participants had different experiences that impact the study.

## Conclusion

The onset of COVID-19 posed multiple challenges to traditional in-person ways of working. Forcing learning to a largely online experience, created difficulties and challenges that occasionally exacerbated existing problems to QI learning. However, despite those, online learning generally worked for action-orientated QI learning, but changes need to be effected to ensure the effectiveness of future use of virtual learning platforms is optimised. For example, healthcare educators may pedagogically, intentionally include more interaction in online learning to facilitate collaboration and demonstrate how learning can be applied. Facilitators need to be aware of and pay attention to the particular challenges that can continue to support QI education through online and hybrid opportunities.

## Electronic supplementary material

Below is the link to the electronic supplementary material.

Supplementary Material 1.

## Data Availability

The data that support the findings of this study are not publicly available under the consent provisions of GDPR and the sensitive nature of the data provided. Anonymized quotation data is available in this paper in line with the consent provisions and further anonymized example quotations in the data set are made available in Table 2. The authors are willing to be contacted to explore reasonable requests for data sharing within the consent provisions at nihr.arc@imperial.ac.uk.

## Abbreviations

ARC NWL: Applied Research Collaboration Northwest London
COVID-19: Coronavirus disease 2019
IC: Imperial College London
NHS: National Health Service
NIHR: National Institute of Health and Care Research
PDSA: Plan, Do, Study, Act
PPI: Patient and Public Involvement
QI: Quality improvement
UK: United Kingdom
