# Supplementary material for: Learning experiences from an online QI fellowship programme during COVID-19 – a qualitative study
Source: BMC Health Serv Res. 2024 Sep 28;24:1144. doi: 10.1186/s12913-024-11590-z (PMC11437894; doi:10.1186/s12913-024-11590-z)
Supplement: Supplementary file 1 — Supplementary Material 1. [file 12913_2024_11590_MOESM1_ESM.docx]

**NIHR ARC Northwest London Improvement Leader Fellowship**

About the Fellowship

The aim of the Fellowship programme is to develop a range of future leaders in innovation and improvement with the authority and ability to advance change in their organisations and beyond, thus building capacity and supporting spread and sustainability in line with the NIHR ARC NWL vision and objectives.

The Fellowship is a 12-month bespoke programme of seminars and workshops with an induction and monthly day-long learning and networking meetings.

The Fellowship uses a collaborative spiral curriculum, where Fellows learn in taught sessions then apply their learning to their project. There is an iterative spiral of learn > apply > learn > apply. The curriculum has three main elements (1) a systematic approach to quality improvement, (2) leadership, and (3) peer-to-peer learning and support.

Fellows are expected to work on their quality/service improvement project with direct relevance to patients and population health benefit.

The Fellowship programme is open to NHS partner organisation staff, academics, people with a role in health and healthcare, and the public. We also welcome Fellows who are patients, carers, or members of the public passionate about changing healthcare.

About the 2021-2022 cohort

In total, there are 17 Fellows, with some working in duos and trios. You can find out more about the Fellows and their projects [here](https://www.arc-nwl.nihr.ac.uk/research/collaborative-learning-and-capacity-building/fellowship/arc-nwl-fellows#h.117qzf4i9dkt).

Focus Group Evaluation questions

- Explore Fellows’ experience of the programme, including what worked well and what worked less well.
- Explore Fellows experience of an implementing improvement projects, including facilitators, barriers, challenges, and highlights.
- Explore the application of learning from the Fellowship, for example:
  - What difference has the Fellowship made to how they work?
  - What difference has the Fellowship made to how they work with their colleagues? Have they shared what they have learned with their colleagues?
  - Aside from their project, where else have they applied the methods and tools they have learned from the Fellowship?
  - How will they use what they learned in the future?
- Explore the impact of Covid on their experience of QI learning and project implementation
  - Did moving online provide any challenges or benefits?
  - What was lost or gained by holding the fellowship online?
